# Helena’s Many Daughters: More Mitogenome Diversity behind the Most Common West Eurasian mtDNA Control Region Haplotype in an Extended Italian Population Sample

**DOI:** 10.3390/ijms23126725

**Published:** 2022-06-16

**Authors:** Martin Bodner, Christina Amory, Anna Olivieri, Francesca Gandini, Irene Cardinali, Hovirag Lancioni, Gabriela Huber, Catarina Xavier, Maria Pala, Alessandro Fichera, Lisa Schnaller, Mario Gysi, Stefania Sarno, Davide Pettener, Donata Luiselli, Martin B. Richards, Ornella Semino, Alessandro Achilli, Antonio Torroni, Walther Parson

**Affiliations:** 1 Institute of Legal Medicine, Medical University of Innsbruck, 6020 Innsbruck, Austria; christina.amory@i-med.ac.at (C.A.); gabriela.e.huber@i-med.ac.at (G.H.); catarinagaxavier@hotmail.com (C.X.); lisa.schnaller@i-med.ac.at (L.S.); 2 Department of Biology and Biotechnology “L. Spallanzani”, University of Pavia, 27100 Pavia, Italy; anna.olivieri@unipv.it (A.O.); gandini.francesca@hsr.it (F.G.); ornella.semino@unipv.it (O.S.); alessandro.achilli@unipv.it (A.A.); antonio.torroni@unipv.it (A.T.); 3 Department of Biological and Geographical Sciences, School of Applied Sciences, University of Huddersfield, Queensgate, Huddersfield HD1 3DH, UK; m.pala@hud.ac.uk (M.P.); alessandro.fichera@perspectum.com (A.F.); m.b.richards@hud.ac.uk (M.B.R.); 4 Department of Chemistry, Biology and Biotechnology, University of Perugia, 06123 Perugia, Italy; cardinali_irene@libero.it (I.C.); hovirag.lancioni@unipg.it (H.L.); 5 Zurich Institute of Forensic Medicine, University of Zurich, 8057 Zurich, Switzerland; mario.gysi@irm.uzh.ch; 6 Laboratory of Molecular Anthropology, Department of Biological, Geological and Environmental Sciences, University of Bologna, 40126 Bologna, Italy; stefania.sarno2@unibo.it (S.S.); davide.pettener@unibo.it (D.P.); 7 Laboratory of Ancient DNA, Department of Cultural Heritage, University of Bologna, 48121 Ravenna, Italy; donata.luiselli@unibo.it; 8 Forensic Science Program, The Pennsylvania State University, University Park, PA 16801, USA

**Keywords:** massively parallel sequencing, next-generation sequencing, forensics, most common haplotype, power of discrimination, mtDNA haplogroup H, random match probability

## Abstract

The high number of matching haplotypes of the most common mitochondrial (mt)DNA lineages are considered to be the greatest limitation for forensic applications. This study investigates the potential to solve this constraint by massively parallel sequencing a large number of mitogenomes that share the most common West Eurasian mtDNA control region (CR) haplotype motif (263G 315.1C 16519C). We augmented a pilot study on 29 to a total of 216 Italian mitogenomes that represents the largest set of the most common CR haplotype compiled from a single country. The extended population sample confirmed and extended the huge coding region diversity behind the most common CR motif. Complete mitogenome sequencing allowed for the detection of 163 distinct haplotypes, raising the power of discrimination from 0 (CR) to 99.6% (mitogenome). The mtDNAs were clustered into 61 named clades of haplogroup H and did not reveal phylogeographic trends within Italy. Rapid individualization approaches for investigative purposes are limited to the most frequent H clades of the dataset, viz. H1, H3, and H7.

## 1. Introduction

Mitochondrial (mt)DNA is a niche marker in forensic genetics that is employed for low copy number and degraded samples, as well as for the investigation of maternal kinship. In these applications, it outperforms nuclear DNA. Yet, its discriminatory power is limited for two principal reasons. The molecule is maternally inherited en bloc, thus even very distant maternal relatives carry identical mtDNAs, barring mutation. In addition, while entire mitogenome sequence data are now more accessible even from forensic samples through massively parallel sequencing (MPS) [1], due to legal and financial restrictions, the current forensic gold standard is to sequence only the ~1.1 kbp of the non-coding mtDNA control region (CR; nps 16024-16569, 1-576) [2] or the ~0.6 kbp of its hypervariable segments HVS-I (nps 16024-16365) and HVS-II (nps 73-340) [3] instead of the entire ~16.6 kbp molecule. Incomplete mitotypes yield higher match probabilities [4] and limit phylogenetic assessment and phylogeographic leads [5]. The distribution of incomplete mitotypes is highly skewed, with a few very frequent ones [4,6]. In West Eurasian populations, the most common mtDNA CR haplotype (MCH) falls into haplogroup H—poetically referred to as “Helena” [7]—and its close relatives. It is characterized by the mutational motif 263G 315.1C 16519C relative to the revised Cambridge reference sequence (rCRS) [8] and is found at a frequency of ~3–4% throughout West Eurasia [9], and in populations of European origin [10], with only slightly lower proportions at the far extensions of West Eurasian populations [11].

The MCH frequency in the high-quality profiles stored in the mtDNA population database EMPOP v4/R13 (https://empop.online, accessed on 15 June 2022) [12] is 4.0%, with a two-sided 95% Clopper–Pearson confidence interval (CI) of 3.7–4.3% in the 15,782 West Eurasians, and 4.7% (CI: 4.2–5.2%) in 8039 European profiles with a minimum CR range. Together with the neighbors carrying exactly one difference, 11.5% of database profiles in EMPOP v4/R13 for West Eurasia and 13.0% for Europe cannot be excluded from deriving from the same maternal lineage or are inconclusive [3,13,14].

The frequency of the MCH is extremely high when compared to autosomal DNA fingerprinting that combines several unlinked loci. It is true that the least powerful STR genotype [15,16] for a single locus of the seven European Standard Set (ESS) core loci [17], viz. TH01 (6|9.3), yields an actual match probability (AMP) of 14.2% in Europe. However, using the high-quality STR allele frequencies stored in the STRidER reference database R2/v2 (https://strider.online, accessed on 15 June 2022) [18], from 7070–7076 individuals genotyped for these markers, the least powerful STR genotype containing all ESS loci (viz. FGA (21|22), TH01 (6|9.3), vWA (17|18), D3S1358 (15|16), D8S1179 (13|14), D18S51 (14|15), D21S11 (29|30)), generates an AMP of 9.8 in 100 million in Europe. Moreover, generally, many more loci than the ESS core set are analyzed, yielding even lower AMPs in STR typing.

The low power of discrimination (PD) for common types is considered the greatest limitation for mtDNA testing [4,19]. A specific mtDNA CR profile, however, does not always anticipate all sequence variation harbored in the complete mitogenome [20]. Phylogenetic CR motifs can predict haplogroup-specific mutations in the coding region (codR; nps 577-16023) but, on the other hand, homoplasy across several haplogroups is common [21] and private variants can never be inferred. Therefore, codR sequencing may allow for mtDNAs with identical CR sequences [4,6] to be distinguished.

In a pivotal study, we collected 29 MCH mtDNAs from Italy and explored their identity in the complete mitogenome. This pilot investigation revealed an extremely high coding region diversity, with only one remaining pair of identical sequences and 28 distinct haplotypes. The discrimination power increased from 0 to 99.8% at the highest resolution and we detected 19 named haplogroup H subclades [9]. To rule out incidental properties of the small dataset and assess the full magnitude of “Helena’s hidden beauty” [9], we here extended the investigation more than sevenfold and present the complete mitogenome sequences from 216 MCH samples in this study, including the 29 initial mtDNAs. We again restricted the donor origin to Italy, where an MCH proportion of 5.6% is reported (see below) to allow for phylogeographic evaluation. Beyond unveiling MCH mitogenome diversity and dispersal in Italy, the data augment the EMPOP etalon of verified mitogenome variation for quality control (QC) and haplogroup estimation [12,22,23].

## 2. Results and Discussion

### 2.1. Mitogenome Diversity behind the MCH

The enormous sequence diversity and almost complete discrimination at the highest level of resolution described for the initial set of 29 Italian MCH mitogenomes [9] was confirmed in the sevenfold extended sample. In the 216 complete mitogenomes identical in the CR, we found 163 distinct haplotypes (Table 1, Table S1).

**Figure 1:**
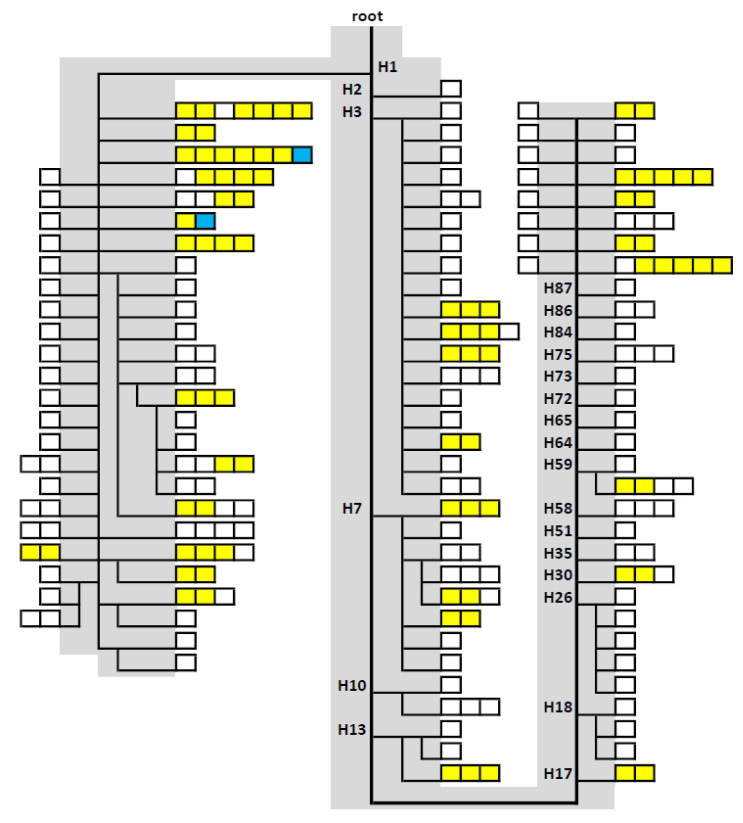
Schematic phylogenetic tree of the 216 mitogenomes sharing the most common West Eurasian CR haplotype motif. The tree is rooted in haplogroup H. The gray backbone and clade names follow PhyloTree*_mt_* Build 17. Every box represents a mitogenome. Adjacent yellow boxes indicate identical mitogenomes. White boxes stand for unique mitogenomes. The blue boxes indicate UniPV_046, which matches two haplotypes with one and six representatives. Stem lengths have no information content (see text and Figure S1 for details).

The statistical assessment of the 216 mitogenomes was complex, since a heteroplasmic individual matched two haplotypes separated by a full difference at that np: UniPV_046, carrying np 6253Y, matched six mitogenomes exhibiting np T6253 and another mitogenome (np 6253C). For calculations, a double match was assumed, resulting in a septet and a pair, both including UniPV_046. Of the 163 haplotypes, 131 haplotypes (80.4%) comprising 60.6% of the samples were unique, resulting in a discrimination capacity (DC) of 75.5%. The 86 non-unique mitogenomes formed 32 groups of identical haplotypes: one septet, two quintets, two quartets, seven triplets, and 20 pairs (Figure 1). We considered alternative scenarios: (i) assuming a total of 217 mtDNAs (as a result of the double match), and (ii) assuming the UniPV_46 matches only with either the sextet (np T6253) or (iii) the singleton (6253C). Other than the obvious changes in the unique and non-unique haplotype statistics, the resulting forensic and population genetic parameters were very similar and differed only at (higher) decimal places between the scenarios (Table S2). Random match probability was 0.9%, and the PD (or haplotype diversity, HD) was 99.6% (Table 1). The increase in the latter among the 216 mitogenomes that were previously considered to be identical from their CR sequence is huge when compared to random West Eurasian population samples consisting of representatives of diverse haplogroups, where the gain was 0.2% in a US “Caucasian” dataset [24] and 1.2% in Basques [25]. Hence, while additional mitogenome sequencing contributes little discriminatory information on randomly mixed population CR datasets in general, in specific cases as described here, its impact can be immense. The most common mitogenome motif among the 216 samples was 263G 315.1C 750G 1438G 3010A 4769G 8860G 15326G 16519C relative to the rCRS [8] with seven representatives (3.2%), two thereof carrying additional point heteroplasmic positions (PHPs), and matching haplogroup H1. While the close maternal relatedness of donors could practically be excluded due to the sampling strategies, 18 of the 32 clusters of identical mitogenomes consisted only of individuals from the same administrative region of Italy, which might indicate some degree of kinship (Table S1). However, simulations show that hundreds of individuals are expected to share identical mitogenomes in a population, and two such individuals are typically a few hundred meioses apart, which corresponds to being “unrelated” for practical purposes [26]. Straightforward methods to identify close kinship in mtDNA population samples have been described [27], but the assessment of more distant relatedness over many generations is laborious [28,29,30] and impossible in a forensic routine setting.

### 2.2. Point and Length Heteroplasmy

We found fifty PHPs, all but one being transitions, in 47 (21.8%) individuals at 47 different nps both in the CR (*n* = 12) and codR (*n* = 35): 146Y, 150Y, 195Y, 204W, 215R (twice), 246Y, 2090R, 2289R, 3003R, 3278Y, 3534Y, 3550R, 3729R, 3943R, 4086Y, 4856Y, 5585R, 6221Y, 6253Y, 6267R, 6716R, 7746R, 7961Y, 8251R, 8252M, 8344R, 8634Y, 9180R (twice), 9828R, 10237Y, 10750R, 11914R (twice), 12373R, 12892Y, 13641Y, 14121Y, 14249R, 14563Y, 14754Y, 14798Y, 15927R, 16080R, 16172Y, 16256Y, 16311Y, 16519Y, and 16527Y. Three samples showed two PHPs, while all other PHPs were the only ones in their sample. Heteroplasmy levels (minor base proportions) ranged from 11–50% (mean 25%, median 24%). They were higher on Ion Torrent platforms (mean 26%, median 25%) than on Illumina platforms (mean 19%, median 18%). The proportion of individuals exhibiting point heteroplasmy was 24.5% on Ion Torrent platforms and 14.2% on Illumina platforms (see below for details on the experiments). Recent MPS population studies found 20.2 [31], 27.5 [32], and 9.0% [25]. Notwithstanding the differences in samples and protocols, the variant detection threshold has a clear influence [33]. One individual (0.5%) carried the heteroplasmic dinucleotide repeat insertion at nps 524.1a 524.2c (note the extended IUPAC nomenclature [2,34]) (Table S1).

### 2.3. Helena’s Many Daughters: Haplogroup Diversity behind the MCH

**Figure 2:**
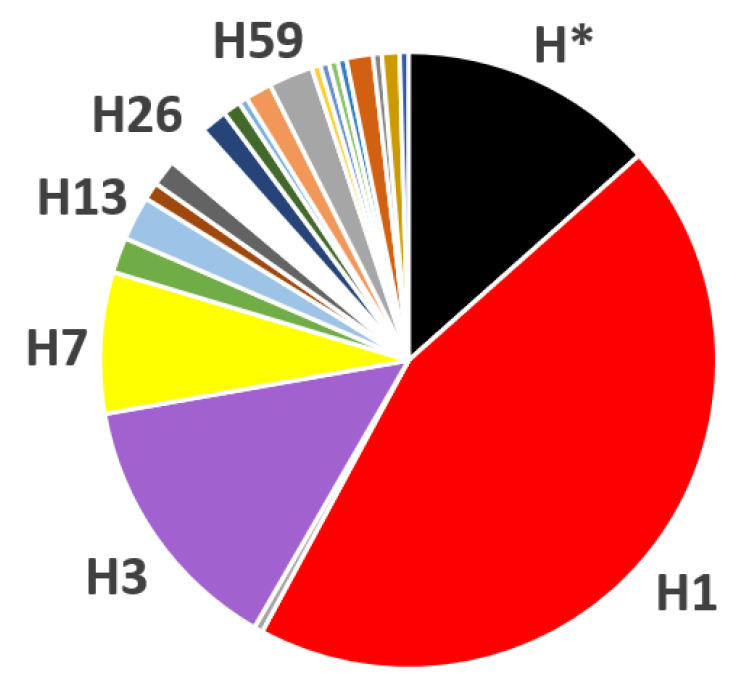
Overview of haplogroups behind the most common West Eurasian CR haplotype revealed by complete mitogenome sequencing (*n* = 216). The proportions of the 22 first-level subhaplogroups of haplogroup H are shown, combining any subclades, and only those with five or more representatives are named, viz. H1 (44.0%), H3 (13.9%), H7 (7.4%), H13 (2.3%), H26 (2.3%), H59 (2.3%), and H* (13.9%). Colors correspond to the colors used in Figure 3. The paragroup H* is included as a single unit (see text and Table 2 for details).

Mitogenome sequencing confirmed haplogroup H status for all 216 mtDNAs. One (0.5%) was found to be an exact haplogroup H representative with no variation in addition to the MCH pattern. A further 29 samples (13.4%) could not be assigned to a named H clade of PhyloTree*_mt_* Build 17 [35]. The remaining 186 mitogenomes were clustered into 60 distinct clades within 22 first-level subhaplogroups at maximum resolution, viz. H1*, H1aj*, H1aj1, H1ax, H1bm, H1bw, H1c2, H1e*, H1e1*, H1e1a*, H1e1a2, H1e2, H1h1, H1j*, H1j3, H1q*, H1q2, H1q3, H1r, H1t, H1u*, H1u1, H1w, H2, H3*, H3ar, H3e, H3q, H7*, H7a, H7b*, H7b1, H7b6, H7c2, H7d3, H7e, H10a, H10c, H13a1a*, H13a1a1, H13a2a, H17, H18*, H18b, H26*, H26a1, H30a, H35, H51, H58, H59*, H59a, H64, H65, H72, H73, H75, H84, H86, and H87 (Table 2, Table S1, Figure S1). The predominant first-level H subhaplogroups in the dataset were: H1, comprising 95 samples (44.0%) in 23 named clades but mainly H1* (43 samples, 19.9%, including seven exact H1 matches); H3 (30 samples, 13.8%) with mainly H3* samples (25 samples, 11.6%, including three exact H3 matches), and further three named clades; H7, whose 16 members (7.4%) were assigned to H7* and seven clades. The 19 remaining rarer first-level H subgroups comprised one to five (mean: 2.4, median: 2) each, and altogether 45 mitogenomes (20.8%) (Figure 1 and Figure 2, Table 2). This confirmed the picture that was yielded from the initial small sample, where H1 (44.8%; including 24.1% H1*), H3 (17.2%; including 13.8% H3*), and H7 (6.9%) were also predominant [9]. Studies agnostic towards a specific CR sequence also revealed H1 and H3 as being predominant H clades in Italy and beyond, peaking in Southwest Europe. Haplogroup H5, also among the top three H clades found in these populations, harbors a CR polymorphism excluding MCH status [36,37,38,39].

### 2.4. Phylogenetic Insights

The findings highlight the importance of research in human mitophylogenetics even after more than four decades and within the most common West Eurasian haplogroup. In addition to numerous singular so-called “private” polymorphisms remaining despite terminal haplogroup assignment found at all clade levels, clusters of related non-identical mitogenomes indicated novel or modified phylogenetic branches within the paraphyletic clusters H*, H1*, and H3*. We did not consider branching solely based on PHPs and polycytosine stretch variation. Several of the shared polymorphism patterns were reported before, intriguingly being mostly from Italy [39,40,41,42] but also Spain [43]. The yet unnamed clusters were H-930A-3531A-4703C (*n* = 3 in this study), H1-15217A (*n* = 7, also in [39]), H1-709A-15470C (*n* = 4), H1-14329T (*n* = 4, also in [39,40,41]), H1e1-11914A-13938T-15930A (*n* = 2, also in [39]), H1q3-@16037-11266T (*n* = 2, also in [42]), H3-6827C (*n* = 4, also in [39,43]), H3-7664A-8406T (*n* = 2), and H3-11200G-(2851G) (*n* = 3, also in [39,40]) (Table S1, Figure S1). Additional unpublished and/or geographically unassigned related mitogenomes are collected in online resources [44,45]. A re-evaluation of signature mutations is emphasized by two further clusters, viz. H1-2851G-12372A-14148G (*n* = 2) and H1e1a-2320G-4823C-(6216C) (*n* = 4), that only partly fulfill the currently described diagnostic pattern for the haplogroups H1h2 and H1e1a5 [35], respectively (see also [9]), and by the uncertain positions of completely sequenced mitogenomes that could be assigned to two clades at similar costs. Here, the most recent common ancestor (MRCA) haplogroups were used [23]: the six representatives of H-3010A-10211T were assigned to H* for H1|H23, and UniPG_033 was assigned to H3* for H3ap|H3ag (Table S1, Figure S1). In a fully resolved phylogeny, any mitogenome sequence will only be assignable to one specific clade and few private polymorphisms will remain [23].

### 2.5. MCH Geography and Phylogeography

**Figure 3:**
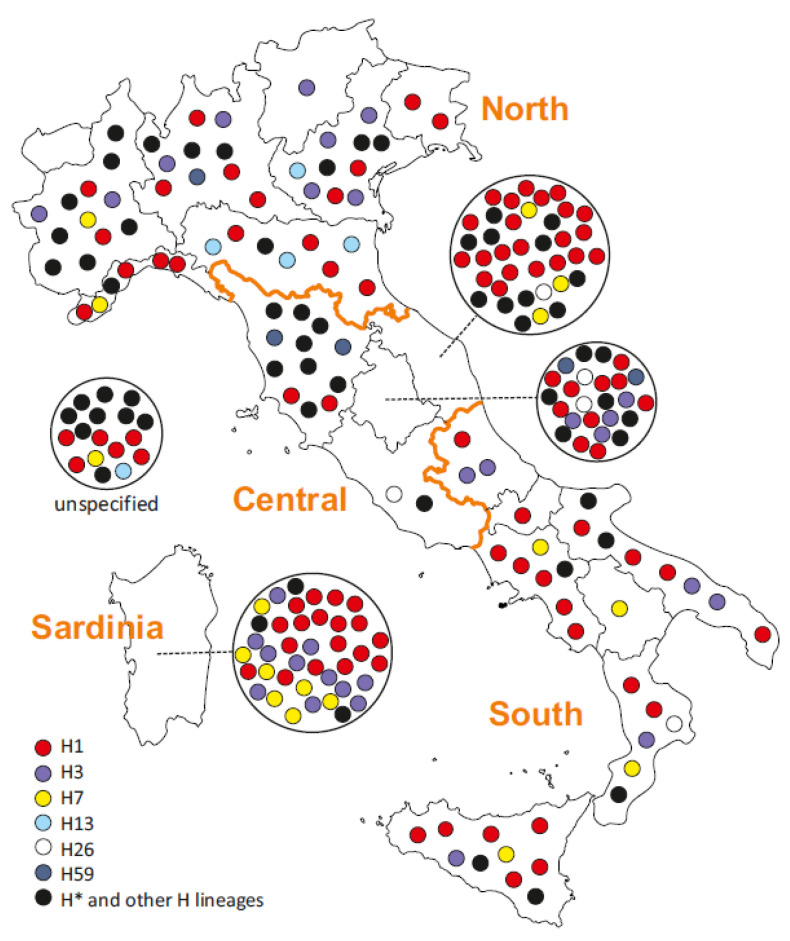
Geographic origin of the Italian individuals whose mitogenomes were analyzed in this study. Circles represent individual mitogenomes. They are assigned to their region of origin for 199 donors, while Italian origin is not further specified for 17 donors. The color codes correspond to Figure 2 and distinguish between the haplogroups H1, H3, H7, H13, H26, and H59 and those falling into H* and other H lineages (see text and Table S1 for details).

The 199 donors with geographic information containing more detail than the national level originated from all the administrative regions of Italy, except for Aosta Valley, the smallest and least populous [46], for which we could not find any published human mtDNA data. The 19 regions were represented by a mean of 10 (4.8%) and median of eight (3.7%) donors each, ranging from one (0.5%) to 38 (17.6%) (Figure 3, Table S1). The geographic dispersal of donor origins results from the foci of collections available at the contributing institutions and does not necessarily reflect the true differences in MCH proportions.

Earlier modern Italian population studies, taken together, reveal little MCH frequency patterns within Italy. Individuals of Italian origin were part of one of the earliest mtDNA sequencing population studies [47] and numerous studies on various geographic scales have been conducted, but even today there are few pan-Italian datasets reporting (at least) the complete CR. The insights are likely biased by the wide range of sampled populations, sample sizes, and sequenced segments. In studies reporting diverse ranges, including both HVS-I and HVS-II data (mostly partial), but less data than the entire CR, the mean proportions of potential MCHs were 8.6% for North [48,49,50,51,52,53,54], 10.1% for Central [42,50,55,56,57,58,59], 7.7% for South Italy [59,60,61,62], and 9.5% for Sardinia [63], resulting in 9.0% over the studies. According to our analyses, the MCH proportion is overestimated from such HVS datasets by one third to one half, mostly due to SNPs in HVS-III (nps 340-576) [2] and np T16519 (unpublished data). Studies covering the entire CR revealed mean MCH frequencies of 6.3% for North [37,38,64], 3.9% for Central [37,38,42,65], 5.5% for South Italy [37,38], and 6.6% for Sardinia [38,39,40], and an overall mean of 5.6%. The latter is similar to the overall results of the datasets covering the entire peninsula (6.3%) [37] or all four macro-areas (6.2%) [38].

### 2.6. MCH Phylogeography and Investigative Implications

**Figure 4:**
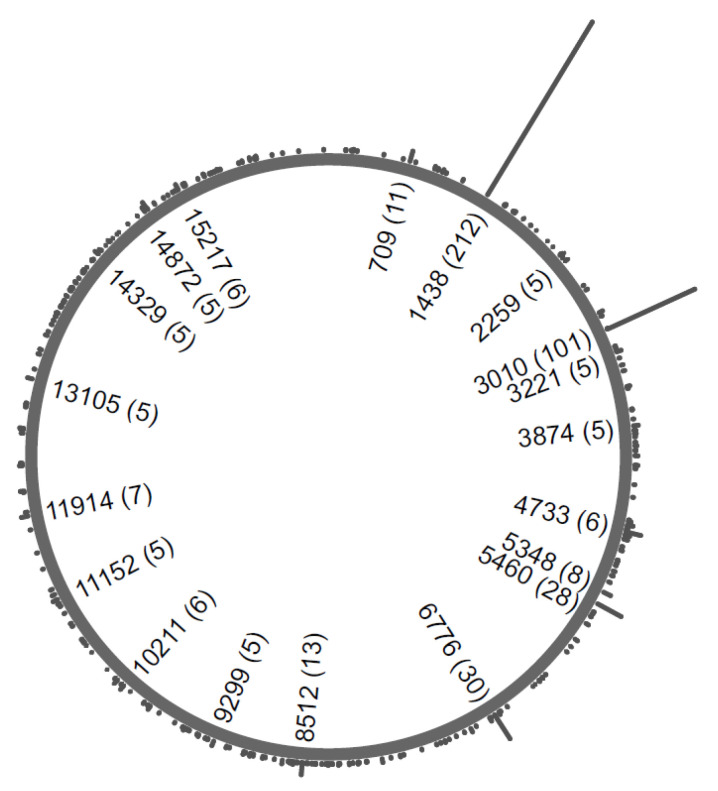
Circular histogram of the variation found in the 216 Italian mitogenomes harboring the most common West Eurasian CR haplotype. Labels were added to nps of the mtDNA molecule where five or more occurrences (indicated in brackets) were observed. Universal variants present in all 216 mitogenomes are not depicted (see text and Table S1 for details).

When plotting the mitogenomes from this study by regional donor origin, the three predominant clades H1, H3, and H7 were found throughout the peninsular and insular regions (Figure 3). Due to the enormous phylogenetic diversity of mitogenomes in this sample set (Figure 1, Figure S1), no other lineages were frequent enough to reveal specific dispersal patterns. The three haplogroups equally ranking fourth in proportion comprised only five individuals each and were geographically restricted, but the patterns were likely caused by the small sample sizes: H13 and H59 were absent in the South, while H26 was absent in the North (Figure 3). Hence, the geographic and phylogenetic distribution of clades behind the MCH over Italy does not seem to contribute investigative leads that would enable the tailoring of the envisioned specific SNP panel for the investigation of the MCH [4,9]. When all the variation found in the 216 mitogenomes was combined, the distribution all over the mitogenome did not reveal “preferred” segments (Figure 4).

Nevertheless, this study has highlighted a promising approach in case an MCH match should be further scrutinized, but complete mitogenome sequencing is not feasible. The screening of diagnostic codR SNPs for the predominant haplogroups H1, H3, and H7 appears to be most effective to investigate differences between the involved MCH mitogenomes (at least in Italy). Typing assays specifically addressing these markers, among others, to circumvent the limited PD of CR have been described [66] and applied in MCH casework [67,68]. When only the three diagnostic markers for H1 (np 3010), H3 (np 6776), and H7 (np 4793) were typed, four haplotypes could be distinguished among the 216 samples reported in this study with 98, 30, 16, and 72 representatives. Accordingly, a random match probability (RMP) of 34.2%, an HD of 66.1%, and a DC of 1.9% would be yielded (Table 1).

## 3. Materials and Methods

### 3.1. DNA Samples

We combined DNA samples donated by Italian residents after informed consent from pre-existing pan-Italian collections of blood, buccal swab, and mouthwash specimens, curated by forensic and population genetic institutions. We considered only the samples with mtDNA sequence information already available for at least partial HVS-I and HVS-II, typically nps 16024-16300 and 73-200, respectively, for this study. The available sequencing data never exceeded CR; sometimes, codR RFLP data was available and always indicated haplogroup H (unpublished). We assessed DNA quantity and integrity in the provided extracts in a modular real-time quantitative assay [69]. We performed Sanger-type sequencing (STS) for CR completion using described protocols [70] and aligned the haplotypes to the rCRS [8] using Sequencer v5.1 (Gene Codes, Ann Arbor, MI, USA). We only included those mtDNAs that exhibited the MCH (263G 315.1C 16519C) from this point. We did not consider heteroplasmy and differences in polycytosine stretch lengths to be preclusive, according to forensic practice [2]. The screening resulted in 187 MCH samples that are collectively presented here for the first time, except for one mitogenome published in advance, in the course of a validation study [31], and 15 partial CR sequences [42]. Together with the pilot sample [9], we investigated a total of 216 mitogenomes in this study. For 17 donors (7.9%), no regional geographic origin information was available. The remaining 199 donors originated from all the administrative regions of Italy except Aosta Valley (Table S1, Figure 3).

### 3.2. Mitogenome Sequencing

Complete mitogenome MPS was performed on Ion PGM (*n* = 61), Ion S5 (*n* = 127) and Illumina MiSeq (*n* = 28) platforms.

For Ion PGM library preparation, we amplified the entire mtDNA molecule as two overlapping ~8.5 kbp fragments [71]. We constructed libraries as previously described [72], quantified using the Ion Library TaqMan Quantitation Kit and normalized to a final concentration of 26 pM. We pooled samples for template amplification and enrichment on the Ion One Touch 2 System (Ion OneTouch 2 and Ion OneTouch ES instruments), using the Ion PGM Template OT2 200 Kit. We loaded the final pool manually onto Ion 314 or 316 chips. Alternatively, for automated template amplification and enrichment, we used the Ion Chef instrument with the Ion PGM Hi-Q Chef Kit. After templating, the samples were automatically loaded on two Ion 316 chips simultaneously for sequencing. We performed sequencing on an Ion PGM using the Ion PGM Sequencing 200 Kit or the Ion PGM Hi-Q Sequencing Kit (all equipment and kits: Thermo Fisher Scientific [TFS], Waltham, MA).

We performed library preparation manually for the Ion S5 using the Precision ID mtDNA Whole Genome Panel with the AmpliSeq Precision ID Library Kit 2.0 or automated using the Precision ID DL8 Kit. For manual library preparation after amplification, we applied the “two-in-one” or “conservative” pooling strategy. We performed partial primer digestion and adapter ligation as described [73]. After library preparation, we quantified all samples using the Ion Library TaqMan Quantitation Kit and normalized them to 30 pM. We pooled samples for template preparation on the Ion Chef. For templating and sequencing, we used either the Ion 520-530 Kit Chef together with the Ion S5 Sequencing Kit or the Ion S5 Precision ID Chef & Sequencing Kit. We sequenced two Ion 530 chips per initialization on an Ion S5 (all equipment and kits: TFS).

We analyzed all Ion PGM data using the Torrent Suite Software suite and the implemented Torrent Mapping Alignment Program to align the raw sequence data in FASTQ format to the rCRS [8]. For variant calling, we used the Torrent Variant Caller plug-in with the default settings of germline low-stringency parameters to generate a variant call format file listing the differences relative to the rCRS in tabular format [72] (all software: TFS). We inspected all the resulting sequences using Integrative Genomics Viewer (IGV) [74] to visualize sequence reads and alignments, to check the consistency of nucleotide calls, and to identify sequencing errors. All Ion S5 data were aligned using Torrent Suite software as described above. More extensive alignment and variant calling for analysis was performed using the HIDGenotyper v2.1 plugin and Converge software v2.1 as described in [75] (both: TFS). Plug-ins were started with default settings. All data were inspected using IGV [74] as described above.

We amplified mtDNA for Illumina MiSeq library preparation as described for the PGM. We quantified the PCR amplicons on an Agilent 2100 Bioanalyzer instrument using the Agilent DNA 12000 Kit (Agilent, Santa Clara, CA, USA) and normalized them to 0.2 ng/µL per amplicon. We prepared libraries using the Nextera XT DNA Sample Preparation Kit according to the manufacturer’s protocol (Illumina, San Diego, CA, USA); after tagmentation (tagging and fragmentation) by the Human mtDNA Genome Sample Prep transposome, we amplified DNA with a limited-cycle PCR program including Nextera XT Index Kit index primers. We cleaned the PCR products using AMPure XP beads (Beckman Coulter, Brea, CA, USA) and normalized them bead- or bioanalyzer-based to 2 nM each. We loaded a 12 pM library pool on the cartridge and sequenced it using the MiSeq Reagent Kit v2 (500 cycles). We inspected all mitogenome sequences and assessed all variants using both the internal MiSeqReporter v2.1 (all: Illumina) with its default variant caller GATK as detailed in [1] and the NextGENe software (SoftGenetics, State College, PA, USA) using the default settings. All data were also inspected using IGV [74] as described above. Four samples were sequenced on an Illumina MiSeq instrument at the Earlham Institute, Norwich, UK following the protocol detailed in [76].

### 3.3. Sequence Data Quality Control

All mitogenome sequences were manually inspected twice by two independent experts and were validated by a third. The relative read depth threshold for variant detection was 10%. We employed further STS and visualization of MPS data in Geneious Prime 2022.0.1 (Biomatters, Auckland, NZ, USA) to clarify doubtful and confirm unobserved variants. For QC purposes, we analyzed a subset of 16 samples (7.4%) independently using two MPS methods with identical sequence results, apart from polycytosine stretch lengths and PHP levels. All results confirmed previous RFLP analyses and STS ([42] and unpublished). All haplotypes passed strict EMPOP QC measures [12].

### 3.4. Haplotype and Haplogroup Assessment

We calculated forensic genetic parameters using Arlequin v3.5 [77] as described [9,66]. In accordance with forensic practice, we disregarded heteroplasmic positions as well as cytosine stretch length variation around nps 309 and 573. We performed calculations and plotting in Microsoft Excel (Office 2019) (Microsoft, Redmond, WA, USA) and the CorelDRAW X7 Graphics Suite (Corel, Ottawa, ON, Canada). We accomplished circular plotting using the genomic visualization software Circos [78] considering all variation in the dataset except for the universal differences at nps 263, 750, 4769, 8860, 16519, and 15326, as well as length heteroplasmy and polycytosine stretch insertions. We counted PHPs as full differences at the np, and block insertions as single events. We assigned indels to the corresponding reference np.

We estimated mtDNA haplogroups from the complete mitogenomes using the SAM2 engine [22] implemented in EMPOP [12], which uses an etalon of verified haplotypes to assess the fluctuation rate of every SNP per clade, instead of following a strict minimal phylogenetic tree classification that considers only the unweighted signature differences but ignores all others. We applied haplogroup names and diagnostic motifs as in PhyloTree*_mt_* Build 17 [35]. Following a conservative approach, we assigned samples with more than one haplogroup candidate producing similar costs to the MRCA haplogroup of the candidates [23].

### 3.5. Published MCH Frequencies

We collected MCH frequencies reported across Italy from published modern population samples. We considered only the datasets covering both HVS-I and HVS-II, at least partially. According to the reported information, we grouped them into a heterogeneous “partial CR” set, when this was the available maximum, and a “full CR” set, when CR or more was available, as well as into the geographic macro-areas of North, Central, South Italy including Sicily, and Sardinia (Figure 3). We split datasets covering more than one area according to geography. Notably, a single publication [38] covered all four areas. The screening resulted in (i) 17 publications containing datasets with partial CR ranges covering North [48,49,50,51,52,53,54], Central [42,50,55,56,57,58,59], South Italy [59,60,61,62], and Sardinia [63]; and (ii) seven reporting at least full CR from North [37,38,64], Central [37,38,42,65], South Italy [37,38], and Sardinia [38,39,40]. The diverse ranges in the “partial CR” category, the heterogeneity of sampled populations, as well as expected inter-laboratory differences in heteroplasmy detection and reporting [79,80], are expected to have introduced some bias in the detected MCH proportions, but not in the interpretations made across datasets. We took data as published, except for correcting the reading frames for mtDNAs LIG15 and MES533 from [37] after personal communication with the authors, and by assuming that 315.1C was omitted and the reported nps 574-576 were truly sequenced, despite violating the stated reading frame in [38].

## 4. Conclusions and Outlook

Earlier studies with limited sample sizes have shown that, after complete mitogenome sequencing, MCH samples rarely match [4,9]. This study presents the largest set of these forensically highly relevant haplotypes compiled from a single country. Extrapolating from the MCH frequency of 5.6% in the published Italian datasets (see above), this sample of 216 MCH mtDNAs represents the screening of 3858 individuals. Applying the West Eurasian and European MCH frequencies in EMPOP [12], the data represent the screening of 5400 (CI: 5023–5837) and 4596 (CI: 4154–5143) individuals, respectively, or roughly one in every 10,000 Italians [46]. This collaborative study shows that, even in a large population sample, random match probability for the MCH is almost zero at the highest resolution. The most common haplotype’s frequency diminishes from 5.6% at the CR (see above) to 0.2% at the mitogenome level, where other haplotypes might be more frequent.

The “*forensic (mito)geneticist’s ultimate desire*” [9] to discern the seemingly identical continues to thrive. Mitogenome sequencing of further common haplotypes would clarify if the same applies also to them. Particularly in other population backgrounds and haplogroups, this approach could help to elucidate the reasons why the MCH is so common among the many star-like haplogroup H clades [9] despite the high CR mutation rate [2]. Contributing factors to the high proportion of this particular haplotype could be an evolutionary or functional constraint or advantage to retain this non-coding sequence, could be a polyphyletic origin, since homoplasy is frequent [21,81] or, plainly, could be the founder effect of the success of haplogroup H representatives (≥40%) in West Eurasian populations [20,36,82] that inevitably makes also their MCH so common [4,6,9]. The ongoing expansion of this study to a West Eurasian scale may reveal patterns of haplogroup dispersal and diversity behind the MCH that were not visible in the investigated single Southern European country and differences in contributing haplogroups. The extended population sample might clarify if the enormous variation reported in this study is a general phenomenon or the consequence of the complex genetic composition of the Italian population, resulting from the large extent, geographic position, and important historic role of the peninsula and the two largest Mediterranean islands, with multiple historic population inputs and migrations across the country, mirrored by both haploid [37,38,42,83,84] and autosomal genomes [85,86,87,88,89,90,91].

## Figures and Tables

**Table 1:** Diversity parameters of the 216 Italian mtDNAs exhibiting the most common West Eurasian control region (CR) haplotype using different sequence ranges. Percentages are rounded (see text for details).

	CR	CR + 3 codR SNPs ^1^	Complete Mitogenome ^2^
Haplotypes	1	4	163
Unique haplotypes	0	0	131
Discrimination capacity (DC)	--	0.019	0.755
Named haplogroups ^3^	1	4	61
Random match probability (RMP)	1.000	0.342	0.009
Power of discrimination (PD) ^4^	0.0%	66.1%	99.6%

^1^ specific for haplogroups H1 (np 3010), H3 (np 6776), and H7 (np 4793); ^2^ see Table S2 for alternative scenarios; ^3^ including the paraphyletic group (paragroup) H*; ^4^ Haplotype diversity (HD).

**Table 2:** Complete list of mtDNA haplogroups found in the 216 most common West Eurasian CR haplotypes at the complete mitogenome level. Bold text indicates first-level subhaplogroups. They comprise any listed subclades. Percentages are rounded.

	*n*	%		*n*	%		*n*	%
**H1**	**95**	**44.0**	**H2**	**1**	**0.5**	**H18**	**3**	**1.4**
H1*	43	19.9	**H3**	**30**	**13.9**	H18*	2	0.9
H1c2	1	0.5	H3*	25	11.6	H18b	1	0.5
H1e*	6	2.8	H3e	2	0.9	**H26**	**5**	**2.3**
H1e1*	2	0.9	H3q	1	0.5	H26*	4	1.9
H1e1a*	9	4.2	H3ar	2	0.9	H26a1	1	0.5
H1e1a2	2	0.9	**H7**	**16**	**7.4**	**H30**	**3**	**1.4**
H1e2	4	1.9	H7*	3	1.4	H30a	3	1.4
H1h1	4	1.9	H7a	1	0.5	**H35**	**2**	**0.9**
H1j*	4	1.9	H7b*	2	0.9	**H51**	**1**	**0.5**
H1j3	2	0.9	H7b1	3	1.4	**H58**	**3**	**1.4**
H1q*	1	0.5	H7b6	3	1.4	**H59**	**5**	**2.3**
H1q2	1	0.5	H7c2	2	0.9	H59*	1	0.5
H1q3	2	0.9	H7d3	1	0.5	H59a	4	1.9
H1r	1	0.5	H7e	1	0.5	**H64**	**1**	**0.5**
H1t	2	0.9	**H10**	**4**	**1.9**	**H65**	**1**	**0.5**
H1u*	3	1.4	H10a	1	0.5	**H72**	**1**	**0.5**
H1u1	1	0.5	H10c	3	1.4	**H73**	**1**	**0.5**
H1w	1	0.5	**H13**	**5**	**2.3**	**H75**	**3**	**1.4**
H1aj*	1	0.5	H13a1a*	1	0.5	**H84**	**1**	**0.5**
H1aj1	1	0.5	H13a1a1	1	0.5	**H86**	**2**	**0.9**
H1ax	1	0.5	H13a2a	3	1.4	**H87**	**1**	**0.5**
H1bm	2	0.9	**H17**	**2**	**0.9**	H*	**30**	**13.9**
H1bw	1	0.5						

## Data Availability

The complete mitogenome sequences are available from GenBank (https://www.ncbi.nlm.nih.gov/genbank, accessed on 15 June 2022) under accession numbers ON597628–ON597814 (novel data) and KM252727–KM252755 (data included in [9]) (Table S1). The information generated in this study will amend the sequence information for the partial mitogenomes already included in EMPOP under accession number EMP00826 [42].

## Supplementary Materials

The following supporting information can be downloaded at: https://www.mdpi.com/article/10.3390/ijms23126725/s1.
